# MicroRNA-138-5p Suppresses Non-small Cell Lung Cancer Cells by Targeting PD-L1/PD-1 to Regulate Tumor Microenvironment

**DOI:** 10.3389/fcell.2020.00540

**Published:** 2020-07-10

**Authors:** Nannan Song, Peng Li, Pingping Song, Yintao Li, Shuping Zhou, Qinghong Su, Xiaofan Li, Yong Yu, Pengfei Li, Meng Feng, Min Zhang, Wei Lin

**Affiliations:** ^1^Institute of Basic Medicine, Shandong Provincial Hospital Affiliated to Shandong First Medical University, Jinan, China; ^2^Institute of Basic Medicine, Shandong First Medical University & Shandong Academy of Medical Sciences, Jinan, China; ^3^Department of Oncology, Shandong Cancer Hospital and Institute, Shandong First Medical University & Shandong Academy of Medical Sciences, Jinan, China; ^4^Departments of Medicine, Tibet Nationalities University, Xianyang, China; ^5^School of Medicine and Life Sciences, Shandong Academy of Medical Sciences, Jinan University, Jinan, China

**Keywords:** non-small cell lung cancer, microRNA-138, dendritic cells, PD-1, PD-L1

## Abstract

Non-small cell lung cancer (NSCLC) is still challenging for treatment owing to immune tolerance and evasion. MicroRNA-138 (miR-138) not only acts as a tumor suppressor to inhibit tumor cell proliferation and migration but also regulates immune response. The regulatory mechanism of miR-138 in NSCLC remains not very clear. Herein, we demonstrated that miR-138-5p treatment decreased the growth of tumor cells and increased the number of tumor-infiltrated DCs. miR-138-5p not only down-regulated the expression of cyclin D3 (CCND3), CCD20, Ki67, and MCM in A549/3LL cells, but also regulated the maturation of DCs in A549-bearing nude mice and the 3LL-bearing C57BL/6 mouse model, and DCs’ capability to enhance T cells to kill tumor cells. Furthermore, miR-138-5p was found to target PD-L1 to down-regulate PD-L1 on tumor cells to reduce the expression of Ki67 and MCM in tumor cells and decrease the tolerance effect on DCs. miR-138-5p also directly down-regulates the expression of PD-L1 and PD-1 on DCs and T cells. Similar results were obtained from isolated human non-small cell lung cancer (NSCLC) cells and DCs. Thus, miR-138-5p inhibits tumor growth and activates the immune system by down-regulating PD-1/PD-L1 and it is a promising therapeutic target for NSCLC.

## Introduction

Lung cancer is the leading cause of cancer-related death in humans (Chen et al., 2011; Saika and Machii, 2012). Non-small cell lung cancer (NSCLC) accounts for approximately 85% of all lung cancer cases (Siegel et al., 2013). Although much progress has been made in the diagnosis and treatment of NSCLC, the mortality of lung cancer remains high (Yang et al., 2013; Smith et al., 2014). Thus, developing molecular targeted treatment approaches for NSCLC is urgently needed.

MicroRNAs (miRs) represent a class short non-coding RNAs. They cause the degradation and/or translation inhibition of respective target mRNAs by directly binding to the 3′-untranslated region (UTR) (Ambros, 2004; Calin and Croce, 2006). A large number of miRs have been associated with various biological processes, including cell survival, apoptosis, proliferation, differentiation, cell cycle progression and migration (Bartel, 2004; Croce and Calin, 2005), participating in the progression of diseases, e.g., cancer (Wen et al., 2015; Mannavola et al., 2016). The effect of miRs on anti-cancer was usually played by directly inhibiting tumor cells growth and/or regulating immune cells to kill tumor cells. MiR-138 has been reported to play a suppressive role in certain common types of human cancer, including brain cancer, osteosarcoma, cervical cancer, larynx carcinoma, and lung cancer, and so on (Sha et al., 2017; Yeh et al., 2019), and is considered to be a promising therapeutic target for cancer. The suppressive function of miR-138 was found to target enhancer of zeste homolog 2 (EZH2) (Zhang et al., 2013; Si et al., 2017), pyruvate dehydrogenase kinase 1 and G protein-coupled receptor 124 (GPR124) (Gao et al., 2014; Ye et al., 2015), SP1 (Liu et al., 2018), SOX9 (Hu et al., 2017), and cyclin D3 (Huang et al., 2015) to inhibit tumor cell growth and migration. Although some studies showed that over-expression of miR-138 in CD4^+^ T cells from psoriasis patients decreased the amounts of Th1/Th2 cells (Fu et al., 2015), and miR-138 in T cells also targeted PD-1 and CTLA-4 to regulate T cell tolerance (Wei et al., 2016). The role and mechanism of miR-138 in the regulation of the tumor micro-environment remains not very clear.

The tumor microenvironment is well known to be immunosuppressive (Zou, 2005; Kim et al., 2006; Rabinovich et al., 2007). Tumor cells consistently release multiple immunosuppressive factors, including vascular endothelial growth factor (VEGF), TGF-β, IL-10, and PGE-2, to facilitate tumor growth and immune escape (Kusmartsev and Gabrilovich, 2006; Shurin et al., 2006; Lin and Karin, 2007). Immune cells in the tumor microenvironment usually are immunosuppressive or tolerant (Todryk et al., 1999; Liu et al., 2009). These immunosuppressive cells include myeloid-derived suppressor cells (MDSCs), Tregs, tumor-infiltrating DCs (TIDCs), and CD11b^*high*^ Ia^*low*^ regulatory DCs (Bell et al., 1999; Li et al., 2008; Liu et al., 2009; Cai et al., 2010). The 3LL lung cancer microenvironment could drive DCs to differentiate into CD11c^ low^CD11b^*high*^Ia^*low*^ regulatory DCs to inhibit T cell response via TGF-β, PGE2, and NO, and so on (Tang et al., 2006; Li et al., 2008; Xia et al., 2008; Liu et al., 2009; Xue et al., 2017). Additionally, high expression of PD-L1 on tumor cells suppresses immune cells via cell-cell contact (Fife et al., 2009; Yokosuka et al., 2012; Chakrabarti et al., 2018; Pawelczyk et al., 2019; Schulz et al., 2019). Inhibiting PD-L1 expression on tumor cells could relieve immune tolerance induced by tumor cells, and blunts tumor cell proliferation (Fife et al., 2009; Topalian et al., 2015; Poggio et al., 2019). How to regulate immune balance in the tumor micro-environment remains a research hotspot.

Herein, the present study aimed to investigate the immune-regulatory mechanisms of miR-138-5p in the NSCLC micro-environment and tumor proliferation to reveal the multi-targeted immuno-modulatory effects of miR-138-5p in anti-cancer therapy.

## Materials and Methods

### Lentivirus Production for miR-138-5p Overexpression

The sequences of human and murine miR-138 were obtained from the National Center for Biotechnology Information database using the Basic Local Alignment Search Tool^1^ and miRBase^2^. The sequence of mature murine miR-138-5p is identical to that of humans. The primer pair of pri-miR-138-5p (sense: 5′ -AG CUGGUGUUGUGAAUCAGGCCGU-3′, antisense: 5′ -GGCCUGAUU CACAACACCAGCUGC-3′) was synthesized by Hanyin Co. (Shanghai, China). The pri-miR-138-5p sequence was cloned into the lentiviral vector PHY-502 carrying green fluorescent protein (GFP) and puromycin sequences by Hanyin Co. (Shanghai, China). Lentivirus which over-express recombinant miR-138-5p (lent-miR-138)and the negative control lentivirus (NC-lentivirus; Hanyin Co., Shanghai, China) were prepared to be 10^9^ TU/ml (transfection unit/ml). To obtain cell lines stably over-expressing miR-138-5p, cells were infected with lent- miR-138, and selected with puromycin (1 μg/ml) for 48 h.

### Animals and Animal Model

Specific pathogen-free C57BL/6 mice and nude mice (approximately 8–10 weeks old, with an average weight of 25 g) were obtained from Beijing Vital River Laboratory Animal Technology Co., Ltd. (Beijing, China). The mice were acclimatized in our animal facility and maintained under specific pathogen-free barrier conditions. All animal experiments were approved by the Animal Care and Use Committee of the Shandong Academy of Medical Sciences.

At day 0, nude mice were inoculated subcutaneously in the right flank with A549 cells labeled with RFP-fluorescent protein (1 × 10^7^ viable cells per mouse in 0.2 ml of DMEM), and randomly divided into three groups, including the tumor (Tumor), lent-NC treatment (Tumor + lent-N), and lent-miR-138-5p-GFP treatment (Tumor + lent-M) groups. After 2 weeks, these mice were administered 1 × 10^8^ PFU/ml NC or miR-138-5p at the tumor location every 4 days. The curative effect was determined by the tumor size, which was measured every 4 days. For the C57BL/6 mouse model, 4–5 × 10^5^ 3LL viable cells/mouse in 0.2 ml of DMEM were subcutaneously injected into C57BL/6 mice. Primary tumor development was monitored by palpation. The largest perpendicular tumor diameters were measured with a caliper at 4-day intervals. Tumor volumes were calculated using the formula π/6 × length × width^2^. Animals were sacrificed by cervical dislocation at day 30 or with subcutaneous tumor volumes exceeding 3,000 mm^3^. When the tumors became palpable with maximum diameter greater than 3 cm at days 10–12, the mice received subcutaneous injections of lent-miR-138-5p-GFP or lent-NC at 4-day intervals for 2 weeks. Control animals received the saline vehicle. *In vivo* live animal imaging was performed on an IVIS (^®^) Lumina III Imaging System (Caliper Life Sciences, Hopkinton, MA, United States). Freshly resected tumor tissue samples were fixed in 4.5% buffered formalin (Th. Geyer, Renningen, Germany) at room temperature for 12–24 h. The fixed tissue was paraffin-embedded, and slides were prepared as previously reported (Zhang et al., 2011; Yang et al., 2017). After dewaxing, the samples were blocked and permeabilized overnight at 4°C in PBS with triton-X100 (1% (w/v) and BSA (10% (w/v). Anti-CD11c or PD-L1 antibodies (Abcam, Cambridge, MA, United States) were diluted in blocking buffer at 4°C for 4 h with gentle shaking. Tissue samples were washed and incubated with secondary antibodies for 2 h before further washing with PBS and incubation with DAPI (10 μM) for 2–3 h.

### Cell Culture

The A549 human cell line was from the Cell Bank of the Chinese Academy of Sciences (Shanghai, China). 3LL Lewis lung carcinoma (clone D122) was a kind gift of professor Chu (Fudan University, Shanghai, China). Cells were cultured in Dulbecco’s Modified Eagle Medium (DMEM) (Gibco BRL, Carlsbad, CA, United States) with 10% fetal bovine serum (Thermo Fisher Scientific Inc., Waltham, MA, United States) at 37°C in a humidified atmosphere containing 5% CO_2_.

Human lung cancer cells were obtained from the tumor tissues of five NSCLC patients undergoing surgery in Cancer Hospital of Shandong Academy of Medical Sciences, after providing signed informed consent. The experiments were approved by the local ethics committee (Cancer Hospital of Shandong Academy of Medical Sciences, Jinan, China). The tumor tissue was homogenized and digested into a single cell suspension with DMEM containing 0.2% collagenase, 0.01% hyaluronidase, and 0.002% DNase at 37°C for 30 min. Then, the tumor cells were isolated with the tumor dissociation kit (Miltenyi Biotec GmbH, Bergisch Gladbach, Germany), according to the manufacturer’s protocol. Human DCs, CD4^+^T cells or CD8^+^ T cells were isolated by Blood dendritic cells isolated Kit II, CD4^+^ T cells or CD8^+^ T cells isolated Kit II (Miltenyi Biotec GmbH, Bergisch Gladbach, Germany).

### Cell Transfection and siRNA Interference

Cells were transfected with adenovirus loaded miR mimics (miR-NC), miR-138 (Genomeditech, Shanghai, China) according to the manufacturer’s instructions.

The PD-L1-specific siRNA sequence (GenBank Accession No. NM_014143) (Zhao et al., 2016) was 5′-GATATTTGCTGTCTTTATA-3′. PD-L1 siRNA and scramble sequences were synthesized and purified by Shanghai Gene-Pharma Co. (Shanghai, China), and transfected into cells with Lipofectamine 2000 (Thermo Fisher Scientific, Inc., Waltham, MA, United States) according to the manufacturer’s instructions.

### Reverse Transcription-Quantitative Polymerase Chain Reaction (RT-qPCR)

Total RNA was extracted using TRIzol reagent (Thermo Fisher Scientific, Inc., Waltham, MA, United States), and reverse transcribed into complementary cDNA with a PrimeScript 1st Strand cDNA Synthesis kit (Takara, Otsu, Japan) according to the manufacturer’s instructions. For mRNA quantitation, qPCR was performed with a SYBR-Green I Real-Time PCR kit (Biomics, Nantong, China) according to the manufacturer’s instructions, with GAPDH as an internal control. The specific primer pairs were as follows: human CCND3 forward, 5′-GAGGTGCAATCCTCTCCTCG-3′ and reverse, 5′-GCTGCTCCTCACATACCTCC -3′; human CDC20 forward, 5′-TGTCAAGGCCGTAGCATGG-3′ and reverse, 5′-AGCACACATTCCAGATGCGA-3′; human MCM2 forward, 5′-ATCTACGCCAAGGAGAGGGT-3′ and reverse, 5′-GCTG CCTGTCGCCATAGATT-3′; human Ki67 forward, 5′-GTTC CAAAAGAAGAAGTGGTGCT-3′ and reverse, 5′-CACAGGCT TCTTTGGAGTAGCAG-3′; GAPDH forward, 5′-CTGGGCTA CACTGAGCACC-3′ and reverse, 5′-AAGTGGTCGTTGAGG GCAATG-3′. Mouse CCND3 forward, 5′- GTGCCCAGGAA ACGGAGTG-3′ and reverse, 5′-CAGCTCCATCCACTGCCAT CAT-3′; mouse CDC20 forward, 5′-CAAATGGAGCAGC CTGGAGA-3′ and reverse, 5′-GACCGTGAACCACTGGATA GG-3′; mouse MCM2 forward, 5′-GGATCTGATGGACAAG GCCAG-3′ and reverse, 5′-AGAGGGTCTGGCCAAGAAGA-3′; mouse Ki67 forward, 5′-AGAGCTAACTTGCGCTGACTG-3′ and reverse, 5′-TTCAATACTCCTTCCAAACAGGC-3′; iNOs forward, 5′-CAATGGCAACATCAGGTCGG-3′ and reverse, 5′-CGTACCGGATGAGCTGTGAA-3′; mVEGF forward, 5′-AGCTACTGCCGTCCAATT-3′ and reverse, 5′-TCTCCGCTCT GAACAAGG-3′; mTGF-β forward, 5′-AAATCAACGGG ATCAGC-3′ and reverse, 5′-TTGGTTGTAGAGGGCAAG-3′; GAPDH forward, 5′-CTGGGCTACACTGAGCACC-3′ and reverse, 5′-AAGTGGTCGTTGAGGGCAATG-3′. The reaction conditions were 95°C for 5 min, followed by 40 cycles of denudation at 95°C for 15 s and annealing/elongation at 60°C for 30 s. The data were analyzed by the 2^–ΔΔ*Cq*^ method.

### Dual-Luciferase Reporter Assay

The human complementary DNA (cDNA) library was used for PD-L1 sequencing and amplification by PCR. The 3′-UTRs of wild-type PD-L1 (PD-L1^*WT*^), and wild-type PD-1 (PD-1^*WT*^), comprising the predicted has-miR-138 binding site, respectively, were cloned into the PHY-811-basic firefly luciferase plasmid (Promega, Madison, WI, United States). Mutant PD-L1 (PD-L1^*mut*^), and PD-1 (PD-1^*mut*^), 3′-UTRs, respectively, were generated with the QuickchangeXL mutagenesis kit (Stratagene, United States) to null the binding of has-miR-138 and cloned into the pGL3-basic plasmid. Then, HEK293T cells were co-transfected with PD-L1^*WT*^ 3′-UTR or PD-L1^*mut*^ 3′-UTR, and Lent-NC or Lent-miR138-5p. Twenty-four hours after transfection, relative firefly luciferase activities were measured with the Dual-Luciferase Reporter Assay System (Promega, Madison, WI, United States) according to the manufacturer’s protocol, normalized to the control with Lent-NC transfection. The interactions of miR138-5p with PD-1, PD-1^*mut*^ were assessed.

### Western Blot

Cells were lysed with ice-cold lysis buffer (Thermo Fisher Scientific, Inc., Waltham, MA, United States), and protein amounts were determined with a Pierce BCA Protein Assay kit (Thermo Fisher Scientific, Inc., Waltham, MA, United States), according to the manufacturer’s protocol. Proteins (50 μg per lane) were separated by 10% SDS-PAGE, followed by transfer onto a polyvinylidene difluoride membrane (Thermo Fisher Scientific, Inc., Waltham, MA, United States). The membrane was then incubated with PBS containing 5% milk at room temperature for 3 h, followed by incubation with rabbit anti-CCND3, CDC20, MCM2, Ki67, PD-1, and PD-L1 primary antibodies (Cell Signaling Technology Inc., Danvers, MA, United States) at room temperature for 3 h. After washing with PBS for 3 times, the membrane was incubated with goat anti-rabbit secondary antibodies (1:5,000, Abcam, Cambridge, MA, United States) at room temperature for 40 min. The samples were then washed three times with PBS, and a Super Signal West Pico Chemiluminescent Substrate kit (Thermo Fisher Scientific, Inc., Waltham, MA, United States) was used to detect signals on an X-ray film according to the manufacturer’s instructions. The relative protein expression was presented as the density ratio vs. GAPDH.

### MTT Assay

3LL or A549 cells (5 × 10^4^ cells/well) were seeded in 96-well plates, and Lent-miR-138 or lent-NC was added for co-culture for 0, 24, 48, and 72 h, respectively. MTT (10 μl, 5 mg/ml, Thermo Fisher Scientific, Inc., Waltham, MA, United States) was added to each well, followed by incubation at 37°C for 4 h. The supernatant was removed, and 100 μl of dimethyl sulfoxide was added per well. Absorbance at 570 nm was determined on a Model 680 Microplate Reader (Bio-Rad Laboratories, Inc., Hercules, CA, United States).

### Flow Cytometry and Antibodies

The tumors were weighed, minced into small fragments, and digested in medium containing 0.1 mg/ml DNase (Sigma-Aldrich) and 1 mg/ml collagenase IV (Sigma-Aldrich) at 37°C for 1 h (Zhang et al., 2011; Yang et al., 2017). The dissociated cells were then prepared for analysis by flow cytometry.

Antibodies targeting CD3ε, CD4, CD8, CD11b, CD80, CD86, CD54, I-a, CD11c conjugated to the corresponding fluorescent dyes were purchased from eBioscience (San Diego, CA, United States). Single-cell suspensions (1 × 10^6^ cells) were stained with different monoclonal antibodies, according to the manufacturer’s instructions. Then, samples were analyzed on a FACSuite using the CellQuest data acquisition and analysis software (BD Biosciences, CA, United States).

### Phenotypic and Functional Identification of DCregs in the Tumor Tissue

Tumor-infiltrating mononuclear cells were isolated from the tumor tissue by Percoll density gradient centrifugation (Zhang et al., 2011; Yang et al., 2017). The obtained cells were labeled with anti-CD11b-PE-cy7, anti-CD11c-FITC, and anti-Ia-PE to analyze the percentage of DCs subsets. DCs was sorted by anti-mouse CD11c Kit (Miltenyi Biotec, Bergisch Gladbach, Germany), CD4^+^ T cells or CD8^+^ T cells was isolated by mouse CD4/CD8 (TIL) microBeads (Miltenyi Biotec, Bergisch Gladbach, Germany) The cells coculture of DC-CD4^+^T cells (1:10), DC-CD8^+^T cells (1:10), or CD8^+^T cells-tumor cells (5:1,10:1, 50:1, or 100:1), DC-tumor cells (10:1) were performed for 72 h, respectively. The effect of DC cells on CD4^+^T proliferation, CTL activation for tumor cell inhibition, and the inhibition on tumors cells were analyzed by flow cytometry with Ki67 and apoptosis detection kit (BD Biosciences, CA, United States).

### Statistical Analysis

Data analysis was performed with GraphPad Prism 5 (GraphPad Software, San Diego, CA, United States). Values are mean ± standard deviation (SD) of three independent experiments. Two-tailed Student’s *t*-test and one-way ANOVA were used as parametric tests. The Mann–Whitney *U*-test and Kruskal-Wallis test were used as non-parametric tests. *P* < 0.05 (^∗^), 0.01 (^∗∗^), and 0.001 (^∗∗∗^) were considered statistically significant.

## Results

### MiR-138 Treatment Inhibits Tumor Growth

**FIGURE 1:**
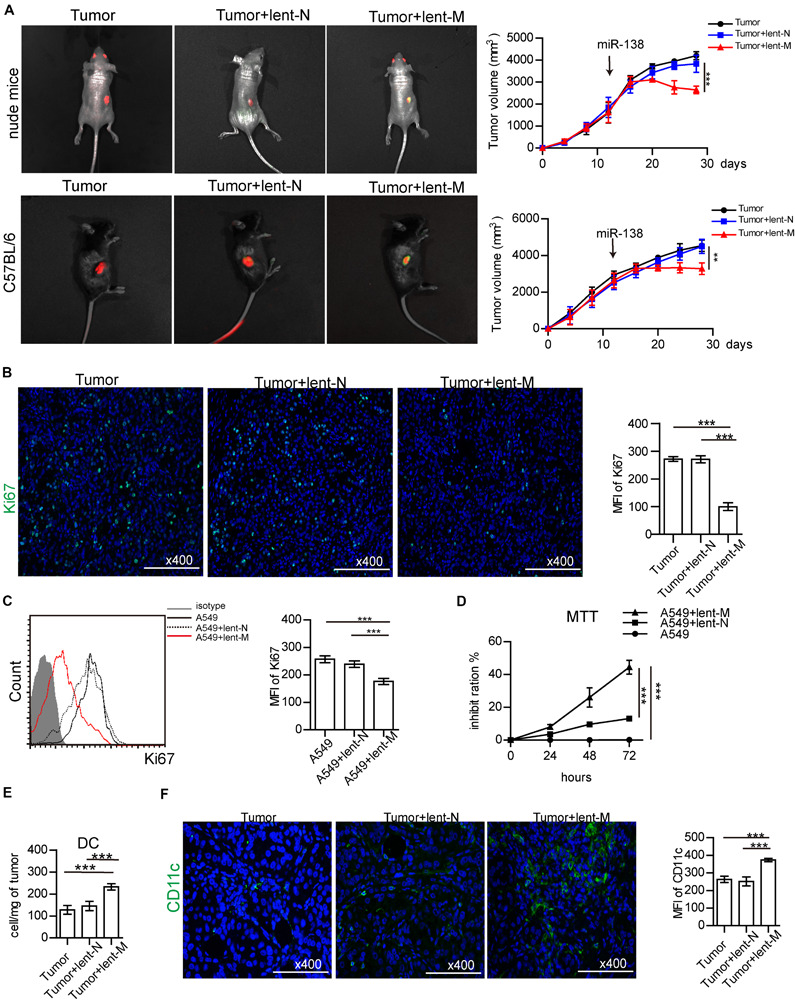
MiR-138 treatment decreases the proliferation of lung tumor cells. **(A)** lent-NC (lent-N, green)/lent-miR-138 (lent-M, green) were administered to nude (left upper panel) or C57BL/6 mice (left bottom panel) bearing corresponding tumor cells (red). After Lent-M treatment, the mean of tumor volume was measured and analyzed (right panel). **(B)** The expression of Ki67 (green) in tumor tissues from lent-M/lent-N treated A549 tumor bearing mice or non-treated animals. (**A,B**, eight mice per group were assessed; ***P < 0.001). **(C)** Flow cytometry detection Ki67 expression on A549 cells, with or without lent-M/lent-N treatment. **(D)** With, lent-M or lent-N treatment or not, the inhibit ratio of the proliferation of A549 cells were measured by MTT. **(E)** The amounts of DCs were elevated in tumors from lent-M treated mice compared with the lent-N-treated and untreated groups. **(F)** Immunofluorescence was used to detect CD11c + cells (green) in tumor tissues from mice treated with lent-M and lent-N, respectively, as well as from untreated animals. The nucleus is blue, Bar = 100 μm. (**C–F**, three independent assays were performed; ***P < 0.001).

To assess the anti-tumor effect of miR-138 in lung cancer, lent-miR-138-5p or lent-N was used to treat the nude mice bearing A549 cells. At 12 days after lent-miR-138-5p treatment, *in vivo* imaging showed lent-miR-138-5p-GFP was accumulated in the area of the tumor expressing red fluorescent proteins. However, lent-NC-GFP did not accumulate in tumor cells (Figure 1A, left upper panel). Similar results were found in mice bearing 3LL tumor cells (Figure 1A, left bottom panel). With lent-miR-138-5p treatment, the tumor volume was decreased (Figure 1A, right upper panel). However, NC treatment did not affect tumor volume (Figure 1A, right upper panel). Similar results were found in C57BL/6 mice bearing 3LL tumor cells (Figure 1A, right bottom panel). Furthermore, Ki67 expression in tumors from miR138-treated-mice was reduced compared with lent-NC-treated- and non-treated mice (Figure 1B). *In vitro* studies showed that Ki67 expression in lent-miR-138-5p-treated-A549 cells was lower compared with lent-NC-treated- and untreated A549 cells (Figure 1C). Meanwhile, the MTT assay confirmed that A549 cell growth was inhibited by lent-miR-138-5p, but not by lent-NC (Figure 1D). Similar results were obtained in 3LL cells (Supplementary Figure S1).

In this study, we found that lent-miR-138-5p treatment increased the amounts of tumor-infiltrating DCs in A549 tumors (Figure 1E). Immunofluorescence showed that there were more CD11c + tumor-infiltrating DCs in the tumor tissue (Figure 1F). The amounts of tumor-infiltrating DCs, CD4^+^ T cells, and CD8^+^ T cells in lent-miR-138-5p-treated 3LL-bearing C57BL/6 mice were also increased compared with these in the other groups (Supplementary Figure S2A). Above all data indicated that miR-138-5p treatment inhibited the proliferation of tumor cells and could alter the immune microenvironment of tumors.

### MiR-138 Treatment Direct Down-Regulates Cell Cycle Related Proteins in Tumor Cells

**FIGURE 2:**
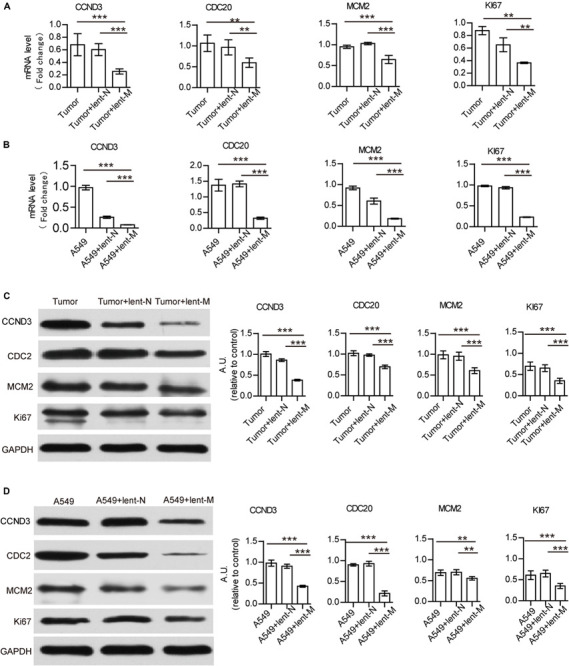
MiR-138 treatment decreases the mRNA and protein expression levels of CCND3, CDC20, MCM2, and Ki67 in A549 tumors and A549 cells. **(A)** Lent-miR-138 treatment (lent-M) decreased the gene expression levels of CCND3, CDC20, MCM2, and Ki67 in tumors from A549 bearing mice, compared with the Lent-NC treated (lent-N) and non-treated tumors. **(B)** Decreased gene expression levels of CCND3, CDC20, MCM2, and Ki67 in lent-miR-138-5p treated A549 cells, lent-NC treated cells and untreated cells. **(C)** Lent-miR-138- treatment decreased the protein expression levels of CCND3, CDC20, MCM2, and Ki67 in A549 tumors or **(D)** A549 cells. (Three independent assays were performed; **P < 0.01, ***P < 0.001).

Furthermore, to analyze the direct effect of miR-138-5p on tumor cells, we detected the mRNA gene expression levels related to growth and immune regulation in tumor tissues by cancer pathway Finder PCR array (Supplementary Table S1). There were four down-regulated proliferation-related genes, which were further detected (Supplementary Table S1). The gene expression levels of Ki67, MCM2, CDC20, and CCND3 in tumor tissues from lent-miR-138-5p treated A549 lung adenocarcinoma tumor-bearing mice were lower than those of the other groups (Figure 2A). The expression levels of Ki67, MCM2, CDC20, and CCND3 were also lower in lent-miR-138-5p treated A549 cells compared with the other groups (Figure 2B). Western blot showed that the expression levels of all these molecules were lower in both lent-miR-138-5p treated A549 tumors and A549 cells compared with the other groups (Figures 2C,D). Lent-miR-138-5p also down-regulated the gene and protein levels of Ki67, MCM2, CDC20, and CCND3 in both lent-miR-138-5p treated 3LL tumors and 3LL cells (Supplementary Figure S3). These data indicated that miR-138-5p had a direct function in inhibiting the proliferation of tumor cells.

### MiR-138-5p Treatment Promotes the Maturation of Tumor-Infiltrating DCs

**FIGURE 3:**
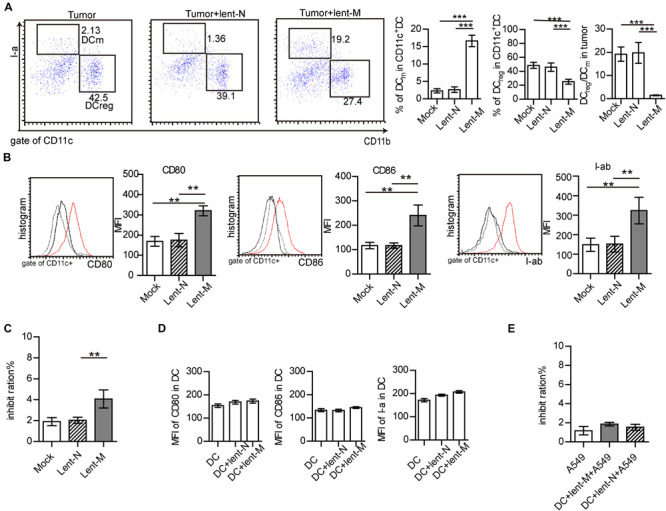
MiR-138-5p treatment changed the statues of DCs in tumor. **(A)** The percentage of mature DCs (DC_*m*_), regulatory DCs (DC_*reg*_) and the ratios of regulator DCs to mature DCs (DC_*reg*_/DC_*m*_) in tumors from A549 bearing mice, which were treated with lent-miR-138 (Tumor + lent-M) or lent-NC (Tumor + lent-N) or not (Tumor). **(B)** The expression levels of CD80, CD86 and I-Ab on tumor infiltrating DCs from lent-M, lent-N treated A549 bearing mice or not. **(C)** The inhibit ration of tumor infiltrating DCs from lent-M, lent-N treated A549 bearing mice or not (mock). Tumor infiltrating DCs were isolated from every group and incubated with A549 cells for 24 h, and the apoptosis ratio of A549 cells was analyzed by flow cytometry. **(D)** With lent-M/lent-N treatment or not, the expression of CD80, CD86, and I-ab on isolated DCs cells. There are no significantly different between every group. **(E)** With lent-M, lent-N treatment, the inhibit ration of DCs on A549 cells were analyzed. There are no significantly different between every group. (**P < 0.01, ***P < 0.001).

Additionally, the immune micro-environment activated by microRNA-138-5p might be another factor for inhibiting the growth of tumors. The statuses of tumor-infiltrating DCs from lent-miR-138-5p treated A549-bearing mice, lent-NC-treated A549-bearing mice, and tumors without treatment were further analyzed. The percentage of I-a^*high*^CD11b^*low*^ (mature DCs, DC_*m*_) in tumor-infiltrating DCs in the microRNA-138 treatment group was higher than those of the other groups (Figure 3A). However, the percentage of I-a^*low*^CD11b^*high*^ (regulatory DCs, DC_*reg*_) in tumor-infiltrating DCs from microRNA-138-5p treatment mice was lower than those of other groups (Figure 3A). The ratio of I-a^*low*^CD11b^*high*^/I-a^*high*^CD11b^*low*^ (regulatory DCs/mature DCs) was decreased in tumor-infiltrating DCs from microRNA-138-5p treatment mice, compared with other groups (Figure 3A and Supplementary Figure S2B). Furthermore, the expression levels of co-stimulatory molecules, including CD80, CD86, and I-ab, were higher on tumor-infiltrating DCs from lent-miR-138-5p treated mice compared with other groups (Figure 3B and Supplementary Figure S2C), indicating enhanced maturity of tumor-infiltrating DCs from lent-miR-138-5p treated A549 bearing mice. The effect of tumor-infiltrating DCs on tumor cells was measured to show that isolated tumor-infiltrating DCs from miR-138-5p-treated nude mice has a higher capability to kill A549 tumor cells/3LL cells (Figure 3C and Supplementary Figure S2D). DCs co-cultured with miR-138-5p-treated 3LL cells has a higher capability to kill miR-138-5p-treated 3LL cells (Supplementary Figure S2E) and promote the killing function of CD8^+^ T cells and the proliferation of CD4^+^ T cell (Supplementary Figures S2F,G).

However, lent-miR-138-5p could not influence the expression of CD80, CD86, and I-ab on isolated DCs in *in vitro* experiments (Figure 3D and Supplementary Figure S4) and could not influence the capability of DCs on A549 cells (Figure 3E), indicating that miR-138-5p could not influence the maturation of DC directly.

### MiR-138-5p Treatment Down-Regulates the Expression of PD-L1 in Tumor Cells, and the Expression of PD-1 on DCs

The maturation of invasive DCs is influenced by many factors, including the secretion of VEGF, TGF-beta, PGE2, eNOS, and IL-10 by tumor cells (Todryk et al., 1999; Kim et al., 2006; Rabinovich et al., 2007; Li et al., 2008; Liu et al., 2009; Cai et al., 2010). The gene expression levels of VEGF, TGF-β, PGE2, and IL-10 in tumor cells from untreated, lent-miR-138-5p treated, and lent-NC treated A549 bearing mice were determined by RT-QPCR. As shown in Supplementary Figure S5A, the gene level of VEGF, TGF-β, PGE2, and IL-10 amounts in the tumors of lent-miR-138-5p-treated mice were similar to those of the lent-NC treated and untreated groups. Similar results were found in lent-miR-138-5p-treated, lent-NC-treated 3LL tumors (Supplementary Figure S5B). Although lent-miR-138-5p treated A549 and 3LL cells decreased the transcription levels of eNOS *in vitro*, the gene level of VEGF, TGF-β, PGE2, and IL-10 in lent-miR-138-5p-treated A549/3LL, lent-NC-treated A549/3LL, and A549 cells/3LL cells were not significantly different (Supplementary Figures S5C,D). This indicated that miR-138-5p may not affect the maturation of dendritic cells through these inhibitory cytokines.

**FIGURE 4:**
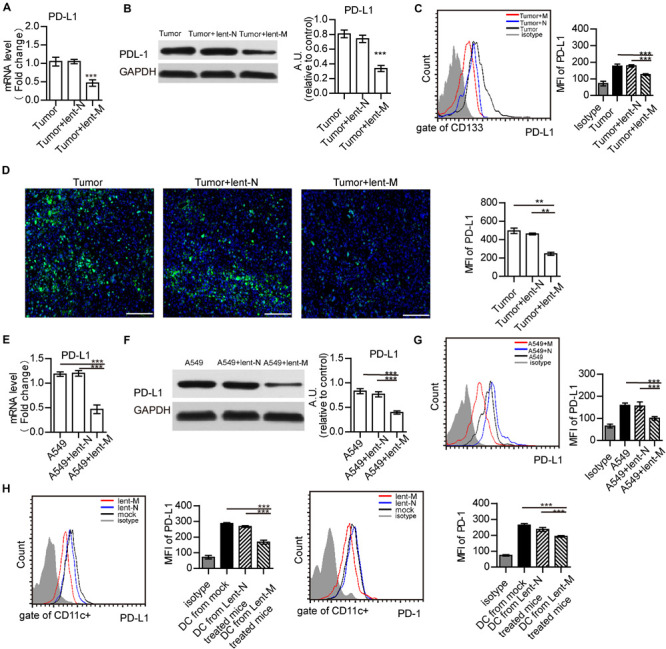
MiR-138 treatment results in decreased expression of PD-L1 in tumor cells. **(A)** The gene expression levels of PD-L1 in tumors from lent-miR-138 (lent-M) treated mice were lower than those of the lent-NC (lent-N) treatment and untreated groups. **(B)** PD-L1 protein expression levels in tumors from microRNA-138 treated mice were lower than those of the NC treatment and untreated groups. **(C)** Flow cytometry analyzed showed that the level of PD-L1 expression on the CD133 + tumor cells from lent-M treated mice was lower than those of the lent-N treatment and untreated groups. **(D)** Immunofluorescence showed that the level of PD-L1 expression on tumors from lent-M treated mice was lower than those with lent-N treatment and untreated groups. Bar = 100 μm (**P < 0.01). **(E,F)** After miR-138 treatment, the gene and protein levels of PD-L1 were decreased in A549 cells. A549 cells were transfected with miR-138 mimic lentivirus (lent-M) or the corresponding negative control (lent-N). Twenty-four hours after lentiviral transfection, RT-qPCR **(E)** or Western-blot **(F)** was performed to examine the gene expression levels of miR-138 in A549 cells (***P < 0.001). **(G)** Flow cytometry analyzed showed that the expression of PD-L1 on the surface of untreated A549 cells, and A549 cells treated with lent-M and lent-N, respectively (***P < 0.001). **(H)** The expression of PD-L1 and PD-1 on the surface of tumor infiltrating DCs from lent-M, lent-N treated A549 bearing mice or not (mock), respectively (***P < 0.001).

PD-L1 is up-regulated in tumor cells and can regulate immune response (Fife et al., 2009; Topalian et al., 2015; Chakrabarti et al., 2018; Pawelczyk et al., 2019; Schulz et al., 2019). Thus, PD-L1 expression in tumor cells was detected. Upon miR-138-5p treatment, the gene and protein expression levels of PD-L1 in the lent-miR-138-5p-treated A549 or 3LL tumor tissue were decreased compared with the other groups (Figures 4A,B and Supplementary Figures S6A,B). Flow cytometry showed that PD-L1 expression decreased on lent-miR-138-5p-treated A549 cells compared with the other groups (Figure 4C). Immunofluorescence of the tumor tissue further proved that PD-L1 expression decreased on lent-miR-138-5p-treated A549 tumor tissue compared with the other groups (Figure 4D). Furthermore, after lent-miR-138-5p treatment, the gene and protein expression levels of PD-L1 on A549 cells were also decreased (Figures 4E–G). Similar results were obtained in 3LL cells (Supplementary Figures S6C,D). Additionally, miR-138-5p treatment decreased the expression of PD-L1 and PD-1 on tumor-infiltrating DCs from lent-miR-138-5p treated mice (Figure 4H), and that on lent-miR-138-5p-treated DCs (Supplementary Figure S7).

### MiR-138 Targets PD-L1/PD-1 to Down-Regulate PD-L1 in Tumor Cells and PD-1 in DCs

**FIGURE 5:**
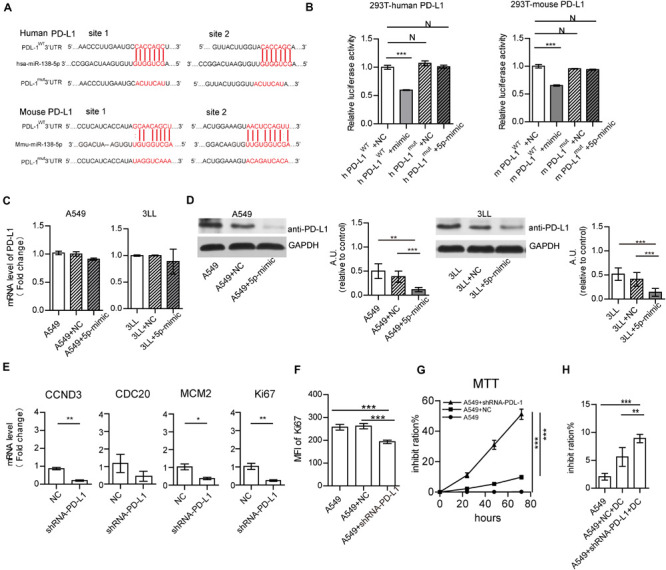
MiR-138-5p targets PD-L1 to decrease proliferation A549 cells. **(A)** Diagram of the has-miR-138-5p binding site in wild-type (WT) human or mouse PD-L1 3′UTR. The mutation was generated at the binding site (Mut). **(B)** In a dual-luciferase reporter assay, a luciferase reporter gene containing the WT or Mut human or mouse PD-L1 3′UTR was co-transfected with NC or miR-138-5p mimic into HEK293T cells, and relative luciferase activities were assessed. The gene **(C)** and protein expression **(D)** of PD-L1 on A549 cells/3LL cells, which were transfected with NC or miR138 mimic or not. **(E)** Upon NC, shRNA PD-L1 treatment, the expression levels of CCND3, CDC20, MCM2, and Ki67 were assessed by RT-QPCR. After NC or shRNA PD-L1 silencing in A549 cells, cell proliferation was analyzed by flow cytometry detection of Ki67 **(F)** or the MTT assay **(G)**. **(H)** Percentages of apoptotic A549 tumor cells co-cultured with DCs, which were pretreated or not with shRNA PD-L1 or NC. (*P < 0.05, **P < 0.01, ***P < 0.001).

**FIGURE 6:**
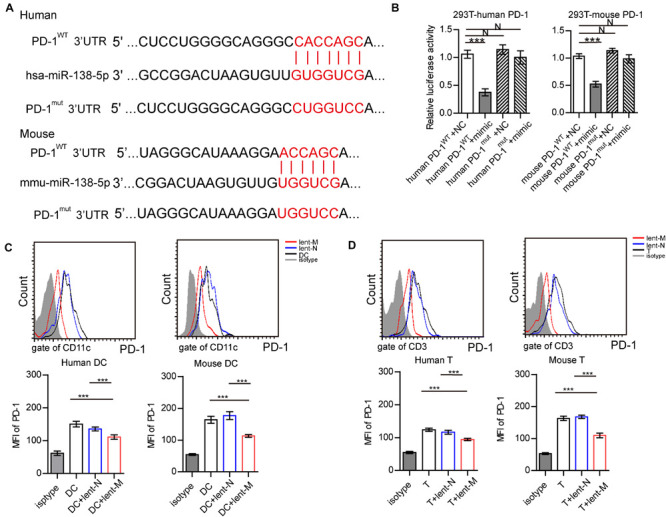
MiR-138-5p targets PD-1 on DCs and T cells. **(A)** Diagram of the has-miR-138-5p binding site on wild-type (WT) human or mouse PD-1 3′UTR. A mutation was generated at the binding site (Mut). **(B)** In a dual-luciferase reporter assay, the firefly luciferase reporter containing the WT or Mut PD-1 3′UTR was co-transfected with NC or miR-138-5p mimic into HEK293T cells, and relative luciferase activities were assessed. After lent-miR-138-5p or lent-NC treatment, the expression levels of PD-1 on human or mouse DCs **(C)** and CD4^+^ T cells **(D)** were determined. (Three independent assays were performed; ***P < 0.001).

Further, the interactions of miR-138 and the *PD-L1, PD-1, eNOS, CDC20, CCND3, Ki67, and MCM2 genes* were analyzed by miRBase^3^ and Targetscan^4^. Besides 3 binding sites were found between human/mouse *CCND3* and miR-138-5p as previous reports (Wang et al., 2012; Han et al., 2016), there are two binding sites between human/mouse *PD-L1* and miR-138-5p (Figure 5A), one binding site between human/mouse *PD-1* and miR-138-5p (Figure 6A). However, there were no direct interactions between miR-138 and *CDC20, Ki67*, or *MCM.* A dual-luciferase reporter assay confirmed that human/mouse *PD-L1* was targeted by miR-138-5p in 293T cells (Figure 5B). To this end, A549 cells were transfected with NC and mimic-miR138-5p, respectively, for 24 h. The gene expression levels of PD-L1 in A549 cells were not significantly different between those groups, but the protein expression levels of PD-L1 in A549 cells/3LL cells were lower than those of other groups (Figures 5C,D). These results demonstrated that miR-138-5p down-regulated the expression of PD-L1 in A549 cells and 3LL cells.

To assess whether PD-L1 affects the proliferation of A549 cells, the PD-L1 gene was suppressed by shRNA PD-L1. PD-L1 silencing did not alter the expression of CDC20 but decreased the gene expression levels of CCND3, MCM, and Ki67 in A549 cells (Figure 5E). This was similar to the effect of miR-138 on 3LL cells (Supplementary Figure S8A). Moreover, PD-L1 silencing resulted in reduced expression of Ki67 in A549 cells/3LL cells and decreased cell proliferation (Figures 5F,G and Supplementary Figures S8B,C). The killing capability of DC on shRNA PD-L1 treated-A549 cells/3LL was higher than those of the no-treatment and NC groups (Figure 5H and Supplementary Figure S8D).

Furthermore, a dual-luciferase reporter assay was performed to confirm that PD-1 was targeted by miR-138-5p in 293 T cells (Figures 6A,B). After lent-miR-138-5p treatment, PD-1 expression levels in human/mouse DCs were lower than those of other groups (Figure 6C). After lent-miR-138-5p treatment, PD-1 expression levels in human/mouse T cells were also lower compared with the other groups (Figure 6D).

### MiR-138-5p Downregulates the Expression of PD-L1 in Human Lung Cancer Cells

**FIGURE 7:**
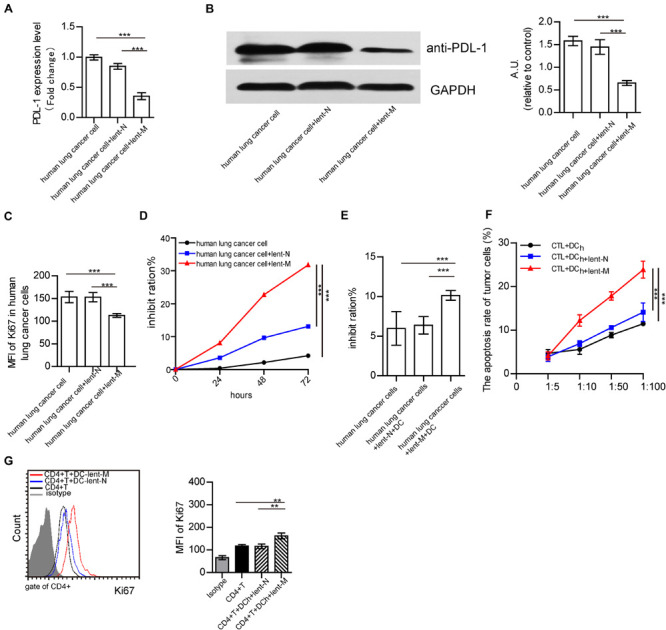
Effects of lent-miR-138-5p on human NSCLC. After treatment with lent-miR-138-5p (Lent-M) or the corresponding negative control lentivirus (lent-N), PD-L1 expression on isolated human lung cancer cells was analyzed by qRT-PCR **(A)** or Western blot **(B)**. Upon lent-miR-138-5p (lent-M) or lent-N treatment, the proliferation of human NSCLC cells was analyzed by flow cytometry detection of Ki67 **(C)** and the inhibition rate of the proliferation of human NSCLC cells was analyzed by MTT assay **(D)**. **(E)** With lent-M, lent-N treatment, the inhibit ration of human DCs on isolated human lung cancer cells were analyzed. **(F)** Percentages of apoptotic tumor cells co-cultured with CD3^+^CD8^+^ human T cells (CTL) activated by human DCs pretreated or not with lent- miR138-5p or lent-N treated A549 cells. **(G)** The proliferation of human CD4^+^ T cocultured with human DCs pretreated or not with lent- miR138 or lent-N treated A549 cells (Three independent assays were performed; **P < 0.01, ***P < 0.001).

To further analyze the effects of microRNA-138 in human lung cancer cells, human lung cancer cells from NSCLC patients were isolated. After lent-miR-138-5p treatment, PD-L1 mRNA, and protein expression levels in human lung cancer cells were decreased (Figures 7A,B). Upon miR-138-5p treatment, the expression of Ki67 in human lung cancer cells was decreased as well as cell proliferation (Figures 7C,D). In addition, lent-miR-138-5p-treated-human lung cancer cells increased the killing capability of DC on tumor cells, compared with the non-treatment group (Figure 7E). Meanwhile, human DCs co-cultured with lent-miR-138-5p-treated-human lung cancer cells had a stronger ability to induce CD8^+^ T cell killing of human lung cancer cells and promote CD4^+^ T cells proliferation (Figures 7F,G).

## Discussion

The expression of miR-138 is reduced in various cancers, including anaplastic thyroid carcinoma (ATC), NSCLC, gallbladder carcinoma, and oral squamous cell carcinoma (OSCC) (Xu et al., 2015; Ye et al., 2015; Li et al., 2016; Hu et al., 2017; Si et al., 2017; Liu et al., 2018). In agreement, miR-138 overexpression can significantly inhibit tumor cell proliferation (Zhang et al., 2013; Li et al., 2015; Ye et al., 2015; Han et al., 2016). Moreover, miR-138 inhibits proliferation, induces apoptosis, restrains both metastasis and invasion, and enhances the chemosensitivity of tumor cells through the suppression of multiple targets (Zhang et al., 2013; Gao et al., 2014; Huang et al., 2015; Ye et al., 2015; Hu et al., 2017; Si et al., 2017; Liu et al., 2018). Thus, miR-138 can play various roles by targeting multiple genes in the biological processes of different cancers and is considered to be a promising molecular target for cancer treatment. MiR-138 also inhibits the proliferation and migration of NSCLC (Zhang et al., 2013; Gao et al., 2014; Ye et al., 2015; Han et al., 2016). However, the mechanism and the regulatory role of miR-138 in the tumor growth and tumor micro-environment remain undefined. Herein, we reported that miR-138-5p could decrease the expression levels of CDC20, CCND3, Ki67, and MCM in tumor cells. However, it only targets CCND3 to inhibit NSCLC, as previously reported (Wang et al., 2012; Han et al., 2016). In our studying, we found that miR-138-5p targets PD-L1 to down-regulate PD-L1 in NSCLC. Silencing PD-L1 reduced the mRNA levels of CCND3, Ki67, and MCM in A549 cells. Thus, miR-138-5p might influence the expression of CCND3, Ki67, and MCM in A549 cells by down-regulating PD-L1. Tumor-intrinsic PD-L1 signaling regulates the distinct tumor cell growth, pathogenesis and autophagy (Chakrabarti et al., 2018; Pawelczyk et al., 2019; Schulz et al., 2019). PD-L1 expression has a positive correlation with Ki-67 expression in glioma (Xue et al., 2017), and its suppression inhibits the proliferation of tumor cells (Topalian et al., 2015; Poggio et al., 2019). Although silencing PD-L1 can not reduce the gene expression of CCD2, lent-miR-138 treatment can decrease the gene expression of CCD2 directly. Thus, the effect of miR-138-5p on the growth of tumors may be through many pathways. Additionally, miR-138-5p treatment decreased the protein expression of PD-L1 in A549/3LL cells, but not the gene expression of PD-L1 in these cells *in vitro*. However, miR-138-5p treatment decreased the gene and protein expression of PD-L1 *in vivo* experiment. It suggested the multiple mechanisms of miR-138-5p on NSCLC, which needs further studying.

Different from previous studies (Song et al., 2011; Wang et al., 2011, 2012; Zhang et al., 2013; Li et al., 2015, 2016; Ma et al., 2015; Xu et al., 2015; Ye et al., 2015; Han et al., 2016; Jiang et al., 2016), assessing the effects of microRNA-138 on tumor cells, our studying showed that miR-138-5p not only inhibits the proliferation of tumor cells but also regulates the tumor immune micro-environment. Lent-miR-138-5p treatment increased the number of tumor-infiltrated DCs and regulated the maturation of DCs in the A549/3LL-bearing mice model, and promoted DCs’ capability to inhibit tumor cells and enhance T cells to kill tumor cells. But lent-miR-138-5p could not induce DC maturation and influence its function directly. Thus, the effect of lent-miR-138-5p on DCs might be through suppress the inhibition of tumor cells or regulate the tumor micro-environment.

The tumor micro-environment is well known to be immune-suppressive. Tumor cells consistently release many immuno-suppressive and pro-inflammatory factors such as vascular endothelial growth factor (VEGF), TGF-β, IL-10, PGE2, eNOS, which induce tumor-infiltrating immune cells to be tolerance (Zhang et al., 2004; Zou, 2005; Kim et al., 2006; Tang et al., 2006; Rabinovich et al., 2007; Xia et al., 2008). Additionally, tumor cells can induce tumor-infiltrating immune cells to be tolerance by PD-L1. In this study, miR-138-5p did not affect the expression of VEGF, TGF-β, IL-10, eNOS and PGE2 in NSCLC cells, while targeting and down-regulating PD-L1, indicating that miR-138-5p could affect the function of DCs by decreasing the expression of PD-L1 on tumor cells. Moreover, down-regulating PD-L1 can reduce the inhibitory effects of tumor cells on DCs to promote CTL–mediated killing of tumor cells and increase the proliferation of T cells. Therefore, miR-138-5p regulates the function of DC by targeting PD-L1. Whether miR-138-5p can regulate the tumor immune micro-environment by targeting other inhibitors need to be further studied.

In cancer patients, miR-138 expression is negatively correlated with the expression of PD-L1 (Huang et al., 2019). PD-L1 is an essential inhibitory molecule in tumor cells that induces tolerance of the tumor micro-environment and immunosuppression. PD-L1 over-expression has been described in different cancers (Topalian et al., 2015; Clark et al., 2016; Chakrabarti et al., 2018; Pawelczyk et al., 2019; Schulz et al., 2019). It promotes immune tolerance in tumors by binding to PD-1 on T cells or DCs to trigger suppressive signaling pathways (Fife et al., 2009; Topalian et al., 2015; Xue et al., 2017; Poggio et al., 2019). Antibody blockade of PD-L1 activates an anti-tumor immune response leading to durable remission in a subset of cancer patients. Blocking PD-L1 expression on tumor cells could be better for tumor therapy and immune activation (Zhao et al., 2016; Poggio et al., 2019). Our studying showed that miR-138-5p targeted PD-L1 to down-regulate PD-L1 in NSCLC and reduce the inhibitory effects on DCs, promote CTL–mediated killing of tumor cells, and increase the proliferation of T cells. Additionally, miR-138-5p also targeted PD-L1 to down-regulate PD-L1 in DCs to relieve the inhibition of tumor-infiltrating DC on T cells. Thus, miR-138 functions not only in inhibiting tumor cell proliferation but also in regulating immune cells by down-regulating PD-L1 on tumor cells.

Similar to previous reports (Wei et al., 2016), miR-138-5p also significantly down-regulated PD-1 in T cells and DCs. PD-1, a receptor of PD-L1, is activated by PD-L1 to induce TCR stopping signals to suppress T cell activation (Parry et al., 2005; Hui et al., 2017). Down-regulating PD-1 can relieve the inhibition of tumor cells by immune cells. Thus, miR-138-5p could regulate immune tolerance by targeting multiple immune checkpoint molecules to inhibit the suppressive cell-cell contact for removing immune-suppression in NSCLC.

Based on these results, our studying showed that the immune-regulatory role of miR-138-5p in the NSCLC micro-environment. This effects of miR-138-5p in anti-cancer therapy were due to miR-138-5p targeting PD-L1/PD-1 to down-regulate the expression of PD-L1/PD-1 (Supplementary Figures S9, S10): (1) miR-138-5p regulated the immune response in the tumor micro-environment by reliving the inhibition of tumor cells on DCs; (2) miR-138-5p inhibited the proliferation of NSCLC cells by decreasing the expression levels of CCND3, Ki67, and MCM2 in tumor cells.

## Data Availability Statement

The datasets presented in this study can be found in online repositories. The names of the repository/repositories and accession number(s) can be found in the article/Supplementary Material.

## Ethics Statement

The experiments were approved by the local ethics committee (Cancer Hospital of Shandong Academy of Medical Sciences, Jinan, China). The patients/participants provided their written informed consent to participate in this study. All animal experiments were approved by the Animal Care and Use Committee of Shandong Academy of medical Sciences.

## Author Contributions

WL designed this research and wrote the manuscript. WL, NS, and PL performed the animal experiments, RT-QPCR, and Western-blot. SZ, QS, YY, XL, PFL, and MF cultured cells and performed the flow cytometry experiment. PS and YL performed the human research experiments. WL and MZ analyzed the data. All authors contributed to the article and approved the submitted version.

## Conflict of Interest

The authors declare that the research was conducted in the absence of any commercial or financial relationships that could be construed as a potential conflict of interest.

## Footnotes

1 http://blast.ncbi.nlm.nih.gov/Blast.cgi

2 http://www.mirbase.org/

3 http://www.mirbase.org

4 http://www.targetscan.org/

## Supplementary Material

FIGURE S1 MiR-138-5p treatment decreases the proliferation of 3LL tumor cells.

FIGURE S2 MiR-138-5p treatment decreases the amounts of tumor infiltrating DCs and T cells from 3LL bearing mice.

FIGURE S3 MiR-138-5p treatment decreases the mRNA and protein expression levels of CCND3, CDC20, MCM2 and Ki67 in 3LL cells bearing mice and 3LL cells.

FIGURE S4 The transduction efficiency of lent-miR-138-5p-GFP in DCs and T cells.

FIGURE S5 MiR-138-5p treatment decreases the mRNA expression levels of VEGF, TGF-β, eNOS, PGE2 and IL-10 in A549 tumor **(A)**, 3LL tumor **(B)**, A549 **(C)** and 3LL cells **(D)**.

FIGURE S6 MiR-138-5p treatment decreases the mRNA and protein expression levels of PD-L1 in 3LL cells.

FIGURE S7 With lent-miR-138-5p treatment, or lent-N treatment, or not, the level of PD-1 and PD-L1 expression on isolated DCs.

FIGURE S8 shRNA PD-L1 influence proliferation of 3LL cell.

FIGURE S9 The design map of our manuscript.

FIGURE S10 The diagram of the effect of miR-138 on tumor cells and DCs.

TABLE S1 With lent-miR138-5p treatment or not, the mRNA expression levels of molecules related to growth and immune regulation in A549 tumor cells by cancer pathway Finder PCR array and showed in table.
