# Corrigendum: MicroRNA-138-5p Suppresses Non-small Cell Lung Cancer Cells by Targeting PD-L1/PD-1 to Regulate Tumor Microenvironment

**DOI:** 10.3389/fcell.2020.00746

**Published:** 2020-08-21

**Authors:** Nannan Song, Peng Li, Pingping Song, Yintao Li, Shuping Zhou, Qinghong Su, Xiaofan Li, Yong Yu, Pengfei Li, Meng Feng, Min Zhang, Wei Lin

**Affiliations:** ^1^Institute of Basic Medicine, Shandong Provincial Hospital Affiliated to Shandong First Medical University, Jinan, China; ^2^Institute of Basic Medicine, Shandong First Medical University & Shandong Academy of Medical Sciences, Jinan, China; ^3^Department of Oncology, Shandong Cancer Hospital and Institute, Shandong First Medical University & Shandong Academy of Medical Sciences, Jinan, China; ^4^Departments of Medicine, Tibet Nationalities University, Xianyang, China; ^5^School of Medicine and Life Sciences, Shandong Academy of Medical Sciences, Jinan University, Jinan, China

**Keywords:** non-small cell lung cancer, microRNA-138, dendritic cells, PD-1, PD-L1

In the published article, there were errors in affiliations 2 and 3. Instead of “Institute of Basic Medicine, Shandong First Medical University & Shandong Academy of Medical School, Jinan, China,” affiliation 2 should be “Institute of Basic Medicine, Shandong First Medical University & Shandong Academy of Medical Sciences, Jinan, China.” Instead of “Shandong Cancer Hospital and Institute, Shandong First Medical University & Shandong Academy of Medical School, Jinan, China,” affiliation 3 should be “Department of Oncology, Shandong Cancer Hospital and Institute, Shandong First Medical University & Shandong Academy of Medical Sciences, Jinan, China.”

The authors apologize for these errors and state that this does not change the scientific conclusions of the article in any way. The original article has been updated.

