# Supplementary material for: MicroRNA-138-5p Suppresses Non-small Cell Lung Cancer Cells by Targeting PD-L1/PD-1 to Regulate Tumor Microenvironment
Source: Front Cell Dev Biol. 2020 Jul 10;8:540. doi: 10.3389/fcell.2020.00540 (PMC7365935; doi:10.3389/fcell.2020.00540)
Supplement: FIGURE S1 — MiR-138-5p treatment decreases the proliferation of 3LL tumor cells. [file Image_1.pdf]

MicroRNA-138 suppresses non-small cell lung cancer cells by targeting PD-L1/PD-1 to regulate tumor microenvironment and inhibit the growth of cancer cells

Nannan Song[1]*, Peng Li[1]*, Pingping Song[2], Yintao Li[2], Shuping Zhou[1], Qinghong Su[1], Xiaofan Li[1], Yong Yu[1], Pengfei Li[3], Meng Feng[1], Zhang Min[3], Wei Lin[1*#]

1 Institute of Basic Medicine, Shandong Provincial Hospital Affiliated to Shandong First Medical University; Shandong First Medical University &Shandong Academy of Medical School, Jinan, 250062, China

2 Department of oncology, Shandong Cancer Hospital and institute, Shandong First Medical University &Shandong Academy of Medical School, Jinan, 250032, China

3 Departments of Medicine, Tibet Nationalities University, Xian City, 712082, China

*co-first author

Correspondence:

Wei Lin, Ph.D.

Institute of Basic Medicine, Shandong Academy of Medical Sciences,

18877# Jingshi Road, Jinan, Shandong province, China, 250032,

Phone:(+86)-0531-82979938,

linw1978@163.com or weilin11@fudan.edu.cn

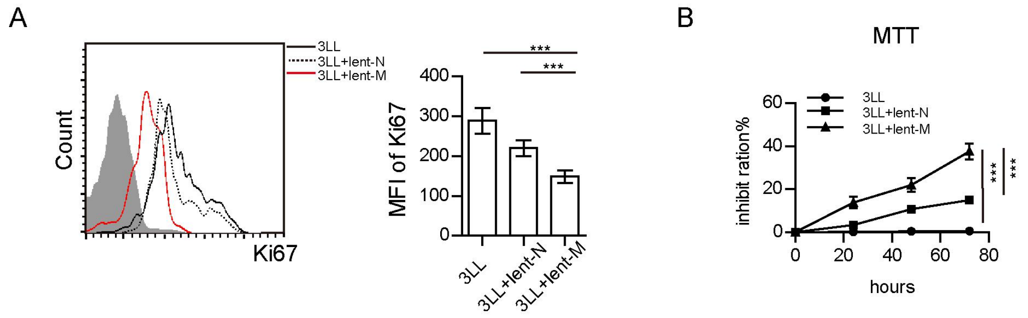

FIGURE S1 | MiR-138-5p treatment decreases the proliferation of 3LL tumor cells. (A) Flow cytometry detection Ki67 expression on 3LL cells, with or without lent-M/lent-N treatment. (eight mice per group were assessed; ***P<0.001). **(B)** After lent-N/lent-M treatment, the inhibit ratio of the proliferation of 3LL cells was analyzed by MTT. (Three independent assays were performed; ***P<0.001).

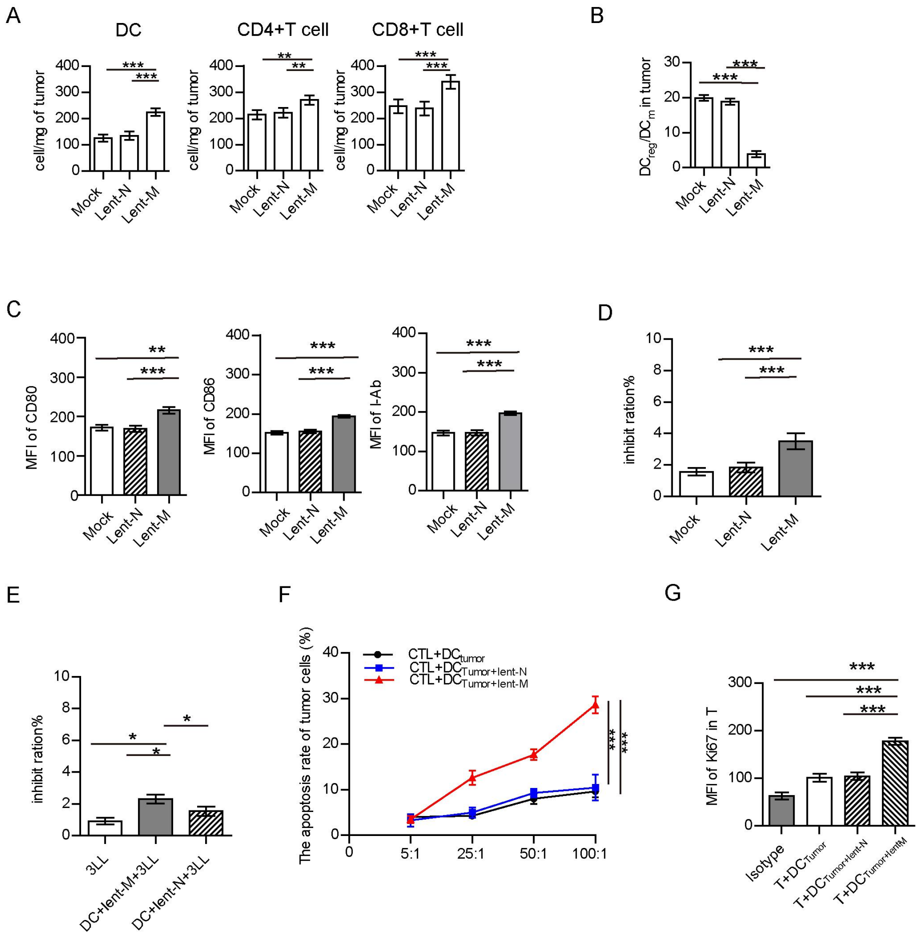

FIGURE S2 | MiR-138-5p treatment decreases the amounts of tumor infiltrating DCs and T cells from 3LL bearing mice. (A) The amounts of DCs and $CD3^{+}CD4^{+}T$, and $CD3^{+}CD8^{+}$ T cells were elevated in tumors from lent-miR-138 treated mice compared with the lent-N-treated and untreated groups. **(B)** Ratios of regulator DCs to mature DCs in tumors from mice treated with lent-miR-138 or lent-N or not. **(C)** The mean fluorescence value of CD80, CD86 and I-Ab expression on tumor infiltrating DCs from 3LL bearing mice treated with lent-M or lent-N or not. **(D)** The inhibit ration of tumor infiltrating DCs, which from lent-M, lent-N treated 3LL bearing mice or not (mock), on 3LL cells. **(E)** The inhibit ration of DCs on lent-M, lent-N treated 3LL cells or not. **(F)** Percentages of apoptosis ratio of tumor cells co-cultured with

$CD3^+CD8^+$ T cells (CTL) which were activated by DCs pretreated with lent-miR138-5p or lent-NC treated 3LL tumor cells or not. **(G)** Upon lent-miR138-5p or lent-N treatment, the proliferation of $CD4^+$ T cells was analyzed by flow cytometry detection of Ki67 (Three independent assays were performed; *P<0.05, **P<0.01, ***P<0.001).

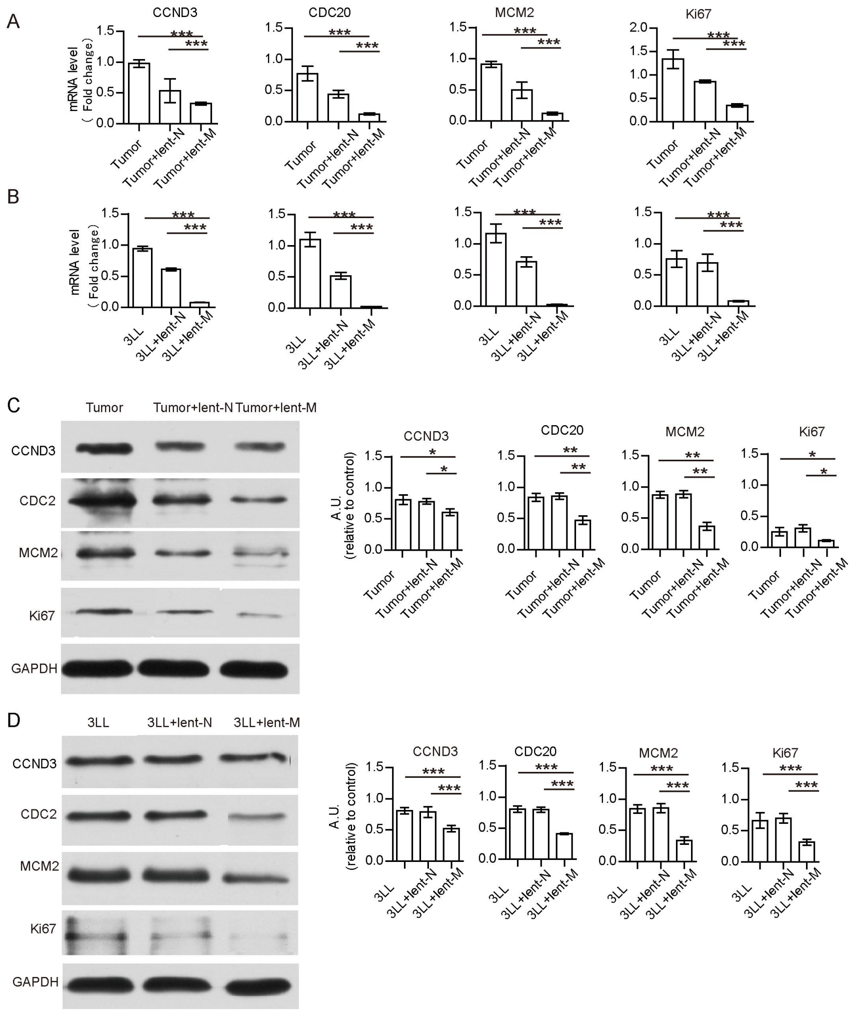

FIGURE S3 | MiR-138-5p treatment decreases the mRNA and protein expression levels of CCND3, CDC20, MCM2 and Ki67 in 3LL cells bearing mice and 3LL cells. (A) Lent-miR-138-5p treatment (lent-M) decreased the gene expression levels of CCND3、CDC20、MCM2 and Ki67 in tumors from 3LL bearing mice, compared with the lent-NC treated (lent-N) and non-treated tumors. **(B)** Decreased gene expression levels of CCND3、CDC20、MCM2 and Ki67 in lent-miR-138-5p treated 3LL cells, lent-NC treated cells and untreated cells. lent-miR-138-5p treatment decreased the protein expression levels of CCND3, CDC20, MCM2 and Ki67 in 3LL tumors **(C)** or 3LL cells **(D)**. (Three independent assays were performed; *P<0.05, **P<0.01, ***P<0.001)

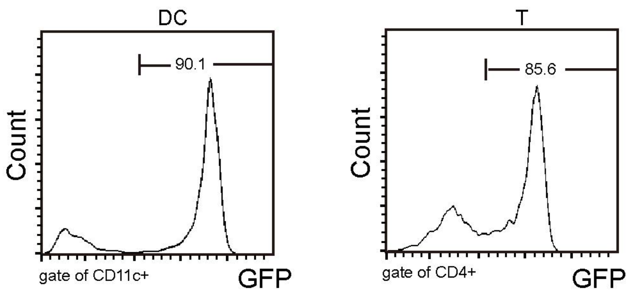

FIGURE S4 | The transduction efficiency of lent-miR-138-5p-GFP in DCs and T cells. The proportion of DC or T cells was transducted by lent-miR-138-5p-GFP.

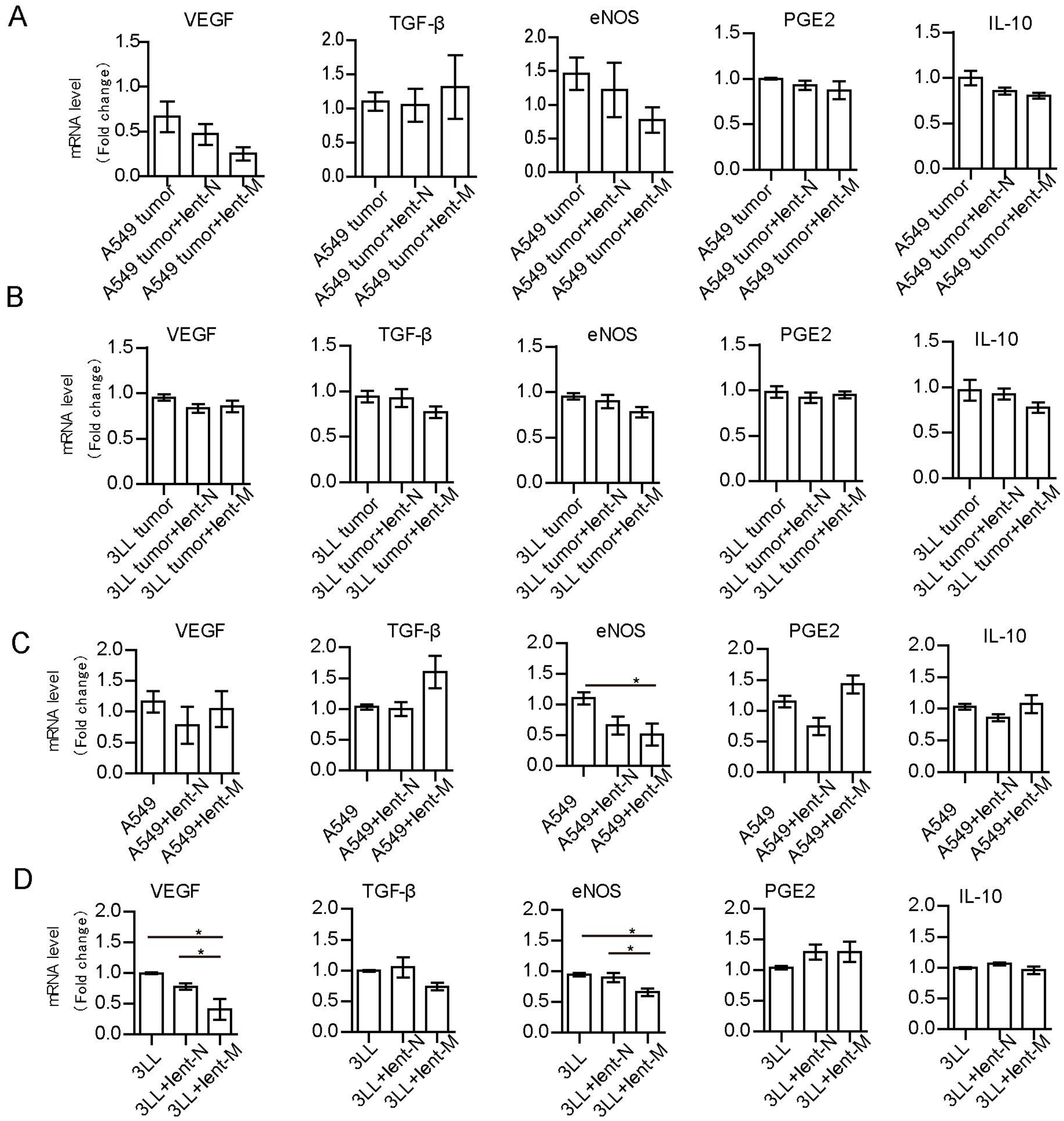

FIGURE S5 | MiR-138-5p treatment decreases the mRNA expression levels of VEGF, TGF-β, eNOS, PGE2 and IL-10 in A549 tumor (A), 3LL tumor (B), A549 (C) and 3LL cells (D) (Three independent assays were performed; *P<0.05, **P<0.01, ***P<0.001).

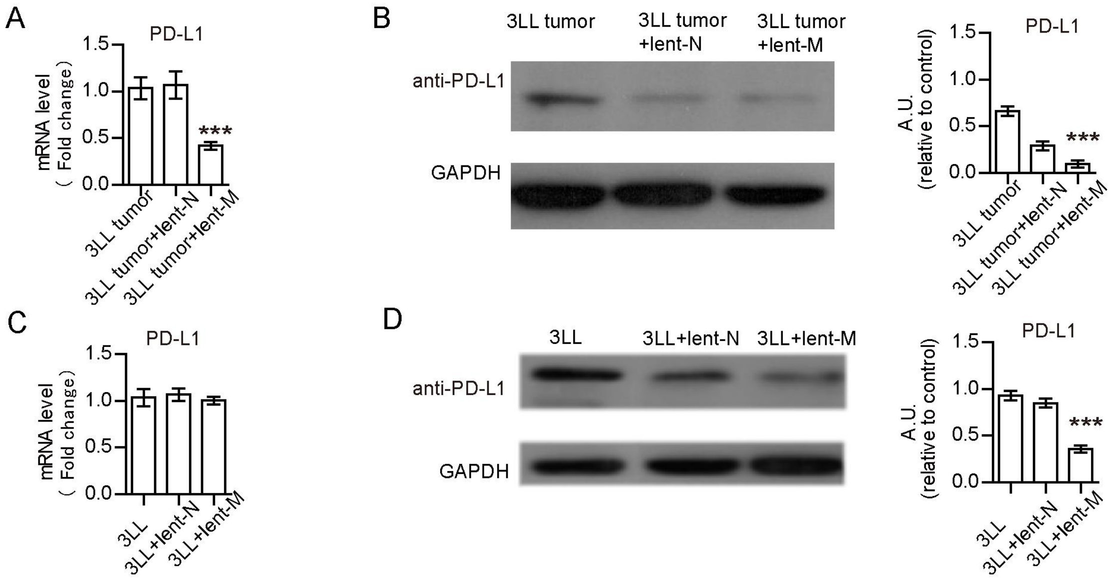

FIGURE S6 | MiR-138-5p treatment decreases the mRNA and protein expression levels of PD-L1 in 3LL cells. MiR-138-5p treatment decreases the mRNA **(A)** and protein expression levels **(B)** of PD-L1 in tumor cells from 3LL bearing mice. Lent-miR-138-5p treatment influence the gene expression levels **(C)** and protein level **(D)** of PD-L1 in 3LL cells (Three independent assays were performed; *P<0.05, **P<0.01, ***P<0.001).

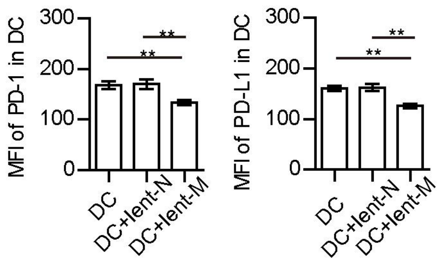

FIGURE S7 | With lent-miR-138-5p treatment, or lent-N treatment, or not, the level of PD-1 and PD-L1 expression on isolated DCs. (Three independent assays were performed; *P<0.05, **P<0.01, ***P<0.001).

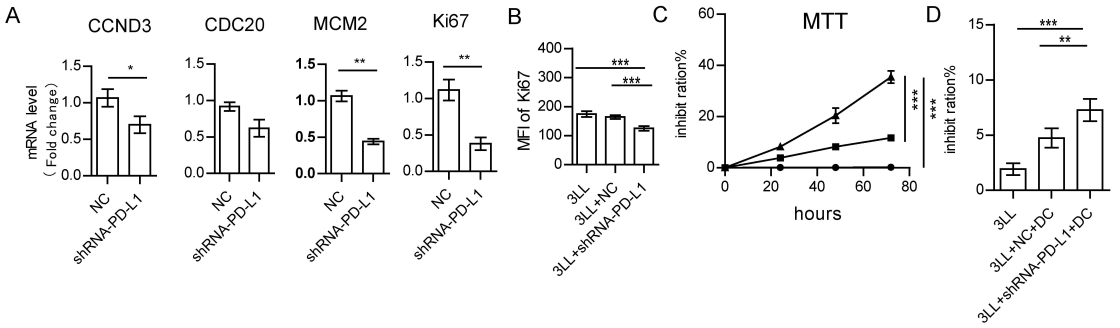

FIGURE S8│shRNA PD-L1 influence proliferation of 3LL cell. (A) shRNA PD-L1 influence the mRNA expression levels of CCND3, CDC20, MCM2 and Ki67 in 3LL cells. With shRNA PD-L1 treatment, the expression of ki67 **(B)** and the inhibit ration of proliferation **(C)** on 3LL cells were measured. **(D)** The killing capability of DC pretreated with shRNA PD-L1 transfected 3LL cells on 3LL cells were analyzed (Three independent assays were performed; *P<0.05, **P<0.01, ***P<0.001).

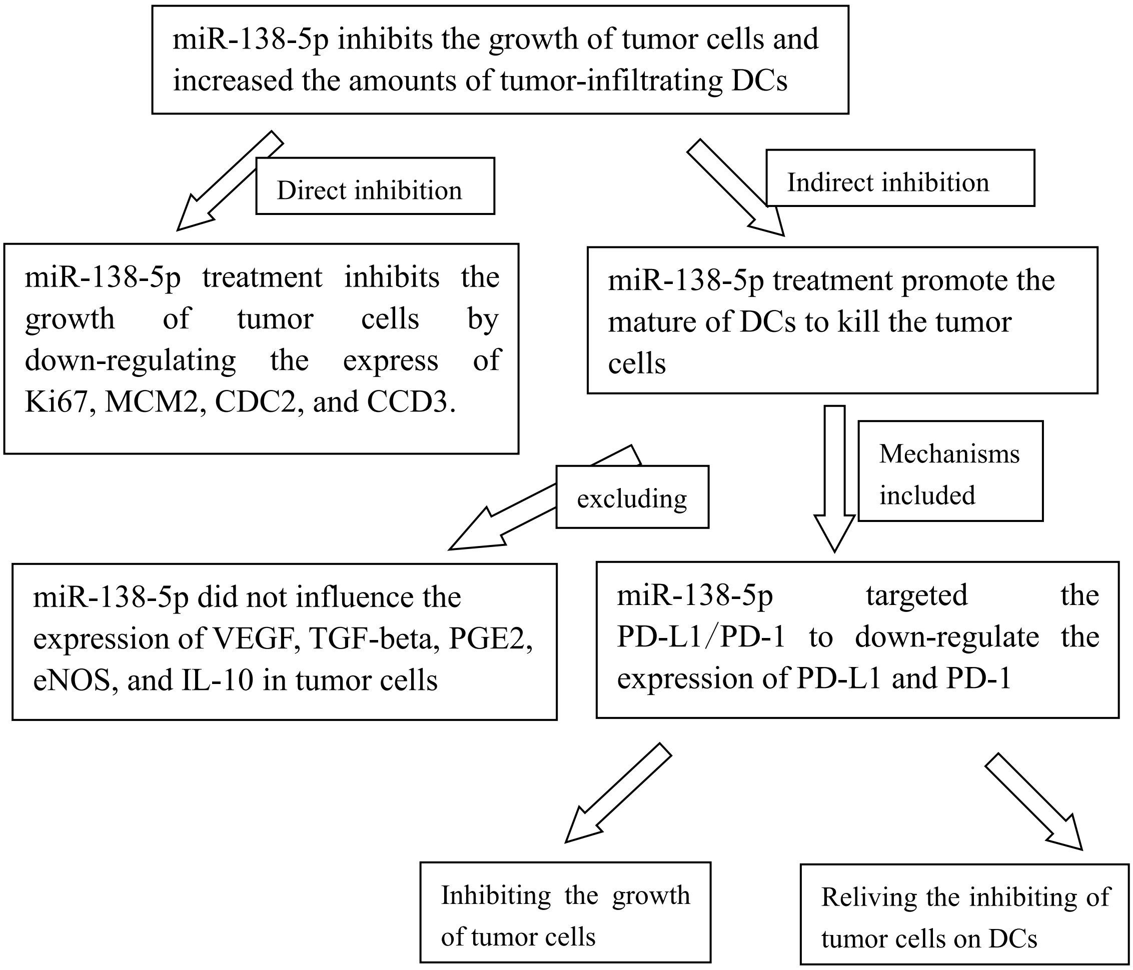

FIGURE S9 | The research roadmap of our manuscript. MicroRNA-138-5p suppresses non-small cell lung cancer cells by targeting PD-L1/PD-1 to relive the inhibition of tumor cells on DCs.

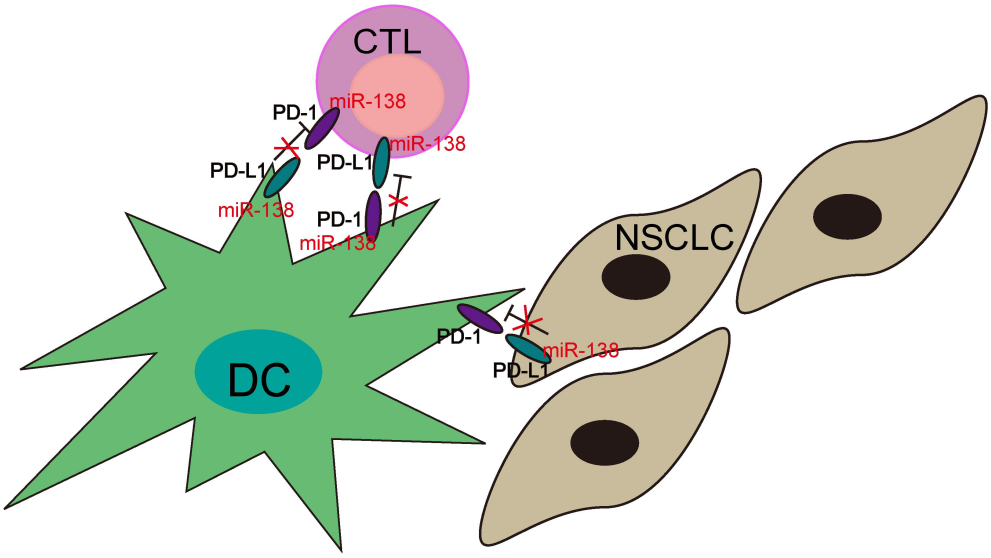

FIGURE S10 | The diagram of the effect of miR-138 on tumor cells and DCs. miR-138 downregulated PD-L1/PD-1 to relive the inhibition of NSCLC cells on DCs, to promote DCs and CTL to kill tumor cells.
