# Supplementary material for: MicroRNA-138-5p Suppresses Non-small Cell Lung Cancer Cells by Targeting PD-L1/PD-1 to Regulate Tumor Microenvironment
Source: Front Cell Dev Biol. 2020 Jul 10;8:540. doi: 10.3389/fcell.2020.00540 (PMC7365935; doi:10.3389/fcell.2020.00540)
Supplement: TABLE S1 — With lent-miR138-5p treatment or not, the mRNA expression levels of molecules related to growth and immune regulation in A549 tumor cells by cancer pathway Finder PCR array and showed in table. [file Table_1.pdf]

MicroRNA-138-5p suppresses non-small cell lung cancer cells by targeting PD-L1/PD-1 to regulate tumor microenvironment and inhibit the growth of cancer cells

Nannan Song[1]*, Peng Li[1]*, Pingping Song[2], Yintao Li[2], Shuping Zhou[1], Qinghong Su[1], Xiaofan Li[1], Yong Yu[1], Pengfei Li[3], Meng Feng[1], Zhang Min[3], Wei Lin[1*#]

1 Institute of Basic Medicine, Shandong Provincial Hospital Affiliated to Shandong First Medical University; Shandong First Medical University &Shandong Academy of Medical School, Jinan, 250032, China

2 Department of oncology, Shandong Cancer Hospital and institute, Shandong First Medical University &Shandong Academy of Medical School, Jinan, 250032, China

3 Departments of Medicine, Tibet Nationalities University, Xian City, 712082, China

Symbol	Fold Change	T-TEST	Fold Up- or Down-Regulation
	G /A	p value	G /A
ACLY	0.98	N/A	-1.02
ACSL4	0.79	N/A	-1.26
ADM	1.09	N/A	1.09
ANGPT1	0.95	N/A	-1.05
ANGPT2	1.27	N/A	1.27
APAF1	1.18	N/A	1.18
ARNT	1.42	N/A	1.42
ATP5A1	0.72	N/A	-1.40
AURKA	0.54	N/A	-1.86
BCL2L11	1.00	N/A	-1.00
BIRC3	0.91	N/A	-1.10
BMI1	0.68	N/A	-1.47
CA9	**0.21**	**<0.01**	**-4.76**
CASP2	0.96	N/A	-1.04
CASP7	0.89	N/A	-1.12
CASP9	0.89	N/A	-1.13

CCL2	1.17	N/A	1.17
CCND2	0.75	N/A	-1.33
CCND3	**0.46**	**<0.05**	**-2.17**
CDC20	**0.42**	**<0.05**	**-2.37**
CDH2	1.97	N/A	1.97
CFLAR	1.72	N/A	1.72
COX5A	1.18	N/A	1.18
CPT2	0.79	N/A	-1.27
DDB2	1.02	N/A	1.02
DDIT3	0.95	N/A	-1.05
DKC1	**0.44**	**<0.05**	**-2.27**
DSP	0.79	N/A	-1.27
E2F4	0.92	N/A	-1.09
EPO	**4.92**	**<0.01**	**4.92**
ERCC3	0.64	N/A	-1.57
ERCC5	1.36	N/A	1.36
ETS2	1.24	N/A	1.24
FASLG	0.77	N/A	-1.31
FGF2	1.16	N/A	1.16
FLT1	**10.01**	**<0.001**	**10.01**
FOXC2	1.28	N/A	1.28
G6PD	0.72	N/A	-1.40
GADD45G	0.57	N/A	-1.77
GPD2	0.81	N/A	-1.24
GSC	**2.59**	**<0.01**	**2.59**
HMOX1	**0.36**	**<0.01**	**-2.78**
IGFBP3	0.89	N/A	-1.13
IGFBP5	1.60	N/A	1.60
IGFBP7	1.19	N/A	1.19
KDR	1.42	N/A	1.42
KRT14	0.52	N/A	-1.91
LDHA	0.85	N/A	-1.18
LIG4	1.19	N/A	1.19
LPL	1.42	N/A	1.42
MAP2K1	0.69	N/A	-1.45
MAP2K3	1.07	N/A	1.07
MAPK14	1.36	N/A	1.36
MCM2	**0.46**	**<0.01**	**-2.15**
MKI67	**0.48**	**<0.01**	**-2.07**
NOL3	1.56	N/A	1.56

OCLN	0.66	N/A	-1.51
PFKL	1.06	N/A	1.06
PGF	0.69	N/A	-1.46
PINX1	0.78	N/A	-1.28
POLB	0.97	N/A	-1.03
PPP1R15A	0.74	N/A	-1.35
SERPINB2	**5.15**	**<0.01**	**5.15**
SERPINF1	1.38	N/A	1.38
SKP2	1.38	N/A	1.38
SLC2A1	**2.09**	**<0.05**	**2.09**
SNAI1	0.80	N/A	-1.24
SNAI2	**5.23**	**<0.01**	**5.23**
SNAI3	**2.49**	**<0.01**	**2.49**
SOD1	0.88	N/A	-1.14
SOX10	**2.10**	N/A	**2.10**
STMN1	0.56	N/A	-1.79
TBX2	1.42	N/A	1.42
TEK	**6.03**	**<0.01**	**6.03**
TEP1	1.90	N/A	1.90
TERF1	0.87	N/A	-1.15
TERF2IP	0.78	N/A	-1.28
TINF2	1.09	N/A	1.09
TNKS	1.24	N/A	1.24
TNKS2	0.77	N/A	-1.31
UQCRFS1	0.70	N/A	-1.43
VEGFC	1.35	N/A	1.35
WEE1	0.69	N/A	-1.46
XIAP	1.12	N/A	1.12
ACTB	0.72	N/A	-1.38
B2M	1.86	N/A	1.86
GAPDH	1.10	N/A	1.10
HPRT1	**0.34**	**<0.05**	**-2.98**
RPLP0	0.68	N/A	-1.47

SUPPLEMENTARY TABLE 1│ With lent-pri-miR138-5p treatment or not, the mRNA expression levels of molecules related to growth and immune regulation in A549 tumor cells by cancer pathway Finder PCR array and showed in table.

Fold-change and fold-regulation values greater than 2 are indicated in red, indicating up-regulated gene; fold-change values less than 0.5 and fold-regulation values less than -2 are indicated in blue, indicating down-regulated gene.p-values: The p values are calculated based on a student's t-test of the replicate 2^(- Delta Ct) values for each gene in the control group and treatment groups, and p values less than 0.05 are indicated in red.
